# In Vitro and In Vivo Evaluation of the Toxic Effects of Dodecylguanidine Hydrochloride

**DOI:** 10.3390/toxics8030076

**Published:** 2020-09-22

**Authors:** Yeon-Mi Lim, Haewon Kim, Seong Kwang Lim, Jean Yoo, Ji-Young Lee, Ig-Chun Eom, Byung-Il Yoon, Pilje Kim, Seung-Do Yu, Ilseob Shim

**Affiliations:** 1 Environmental Health Research Department, National Institute of Environmental Research, Incheon 22689, Korea; ymlim@korea.kr (Y.-M.L.); hwkim91@korea.kr (H.K.); lsgwang11@korea.kr (S.K.L.); Jeanyoo@korea.kr (J.Y.); eeasy@korea.kr (J.-Y.L.); iceom@korea.kr (I.-C.E.); newchem@korea.kr (P.K.); sydu@korea.kr (S.-D.Y.); 2 College of Veterinary Medicine and Institute of Veterinary Science, Kangwon National University, Chuncheon 24341, Korea; byoon@kangwon.ac.kr

**Keywords:** dodecylguanidine hydrochloride, guanidine-based chemicals, inhalation toxicity, bronchoalveolar lavage, inflammation

## Abstract

The toxicity profiles of the widely used guanidine-based chemicals have not been fully elucidated. Herein, we evaluated the in vitro and in vivo toxicity of eight guanidine-based chemicals, focusing on inhalation toxicity. Among the eight chemicals, dodecylguanidine hydrochloride (DGH) was found to be the most cytotoxic (IC_50_: 0.39 μg/mL), as determined by the water soluble tetrazolium salts (WST) assay. An acute inhalation study for DGH was conducted using Sprague-Dawley rats at 8.6 ± 0.41, 21.3 ± 0.83, 68.0 ± 3.46 mg/m^3^ for low, middle, and high exposure groups, respectively. The levels of lactate dehydrogenase, polymorphonuclear leukocytes, and cytokines (MIP-2, TGF-β1, IL-1β, TNF-α, and IL-6) in the bronchoalveolar lavage fluid increased in a concentration-dependent manner. Histopathological examination revealed acute inflammation with necrosis in the nasal cavity and inflammation around terminal bronchioles and alveolar ducts in the lungs after DGH inhalation. The LC_50_ of DGH in rats after exposure for 4 h was estimated to be >68 mg/m^3^. Results from the inhalation studies showed that DGH was more toxic in male rats than in female rats. Overall, DGH was found to be the most cytotoxic chemical among guanidine-based chemicals. Exposure to aerosols of DGH could induce harmful pulmonary effects on human health.

## 1. Introduction

Various chemicals are widely used as active ingredients in consumer products, functioning as a preservative, disinfectant or antimicrobial agents [1,2]. With the number of such chemicals increasing rapidly, it has become difficult to evaluate their toxic effects on human health. Currently, the use of consumer products is highly prevalent and a large number of people are routinely exposed to these products over a short period of time, thus increasing the concern about their harmful effects on human health [3].

In the Republic of Korea, exposure to humidifier disinfectants was identified as the potential cause of an outbreak of idiopathic lung injury in 2011. It was reported that over 8 million people were exposed to humidifier disinfectants [4] resulting in over one thousand deaths until 2017 [5]. The reason for the public health crisis due to these consumer products could be that some chemicals that are considered nontoxic through oral intake and dermal exposure might be seriously toxic if they are inhaled [2]. Unfortunately, humidifier disinfectants, which have been reported to cause lung injury, were on the market without safety assessment for inhalation [6].

Polyhexamethylene guanidine (PHMG), oligo(2-(2-ethoxy)-ethoxyethyl)guanidinium-chloride (PGH), and methylchloroisothiazolinone/methylisothiazolinone (CMIT/MIT) are the main constituents of humidifier disinfectants [7]. PHMG and PGH are well-known disinfectants and cationic polymer chemicals synthesized by guanidine-based chemical groups. The commonly known characteristics of guanidine are attributed to the functional group of the arginine side chain, which is a strong base and behaves as a cation over a wide pH range. In addition, it is known that the guanidine group interacts with biological molecules with carboxylic and phosphate groups through ion-pair formation, using electrostatic interactions and hydrogen bonding [8]. Compounds that include a guanidine moiety have been isolated from various microorganisms and have been shown to be biologically active, either as antimicrobial, antiviral, or antifungal agents [9,10]. Many guanidine-based chemicals exert antimicrobial activity because guanidine can interact with the phosphate group of the phospholipid membrane. The main antimicrobial mechanism of guanidine- based chemicals is the destruction of the cytoplasmic membrane of bacterial cells [11,12,13,14,15]. Owing to this property, guanidine-based biocidal products are commonly used in various consumer products, and thereby humans are routinely exposed to these chemicals.

The purpose of the present study was to evaluate the toxicological effects of guanidine-based chemicals through both in vitro and in vivo experiments. We first searched for guanidine-based chemicals that are distributed commercially in large quantities, through the national chemical database: the National Chemical Information System (https://ncis.nier.go.kr). Eight guanidine-based chemicals were selected and analyzed using the cell viability assay as an in vitro screening step for the subsequent inhalation toxicity study. Next, Sprague-Dawley (SD) rats were exposed to the aerosol of dodecylguanidine hydrochloride (DGH), a guanidine-based chemical, which was found to have the highest cytotoxicity in the in vitro assays. Results from this study confirmed that exposure to aerosols of DGH was detrimental and exerted harmful pulmonary effects in rats.

## 2. Materials and Methods

### 2.1. Chemicals

Dodecylguanidine hydrochloride (DGH) was purchased from Lanxess Co. (Cologne, Germany). Cyanoguanidine polymer, with ammonium chloride and formaldehyde, (CGP) was purchased from Leap LabChem (Zhejiang, China) and guanidine sulfamate (GS) was obtained from TCI Chemicals (Tokyo, Japan). Other chemicals (1,1,3,3-tetramethylguanidine (TMG), guanidine carbonate (GC), guanidine monohydrochloride (GH), guanidine mononitrate (GN), and cyanoguanidine (CG) were purchased from Sigma-Aldrich (St. Louis, MO, USA).

### 2.2. Cell Culture

Human adenocarcinomic alveolar epithelial (A549) cells were obtained from the Korea Cell Line Bank (Seoul, Korea). Cells were grown in RPMI 1640 medium (Thermo Fisher Scientific, Waltham, MA, USA), containing 10% heat-inactivated fetal bovine serum (Thermo Fisher Scientific, Waltham, MA, USA) and 1% penicillin-streptomycin (Thermo Fisher Scientific, Waltham, MA, USA) under standard cell culture conditions (37 °C, 5% CO_2_, and 90% humidity).

### 2.3. Evaluation of Cytotoxicity

A549 cells (1 × 10^4^ cells) were incubated in 96-well plates overnight, and various concentrations (1, 10, 100, 1000, and 5000 μg/mL) of the eight selected chemicals were added to the cell culture medium. After 24 h of incubation, cell morphology was observed by optical microscopy, and cell viability was evaluated using EZ-Cytox kit (DoGenBio, Seoul, Korea) by quantifying purple formazan crystals. The membrane integrity was examined using an EZ-lactate dehydrogenase (LDH) kit (DoGenBio, Seoul, Korea) to measure the LDH level released from the cytosol into the culture medium. Absorbance was measured using a microplate reader (Tecan, Salzburg, Austria) at a wavelength of 450 nm.

### 2.4. Colony Formation Assay

A clonogenic assay was performed by modifying the method of Franken et al. [16]. Briefly, the cells were incubated overnight in six-well plates, at a density of 200 cells per well. DGH (0.00313, 0.00625, 0.0125, 0.025, 0.05, 0.1, and 0.2 μg/mL) and CGP (0.313, 0.625, 1.25, 2.5, 5, 10, and 20 μg/mL) were incubated with the cells for ten days. After incubation, the medium was removed, the cells were washed with phosphate-buffered saline (PBS), fixed with methanol for 10 min on ice, and stained with crystal violet (0.5% crystal violet in 20% methanol). The number of colonies was quantified using light microscopy (Olympus Co., Tokyo, Japan).

### 2.5. Experiment Animals

Specific-pathogen-free (SPF) male and female Sprague-Dawley (six-week-old) rats were purchased from Orient Bio Inc. (Sungnam, Korea). The temperature and relative humidity of the animal maintenance room was set to 22 ± 3 °C and 30 to 70%, respectively, with a 12:12 h light/dark cycle. Diet (LabDiet 5053, Orient Bio Inc., Sungnam, Korea) and water were supplied ad libitum. Following an acclimatization period of one week, the rats were randomly divided into four groups (five males and five females per group) for the acute inhalation study. All animal experiments were approved by the Animal Care and Use Committee of the National Institute of Environmental Research, Republic of Korea (identification code: NIER-2019-01-01-011; date of approval: 15 May 2019). The left lung of the rats was used for histopathological analysis and the right lung was used for bronchoalveolar lavage fluid (BALF) analysis, including lung cell damage and pulmonary inflammation.

### 2.6. Inhalation Exposure to Dodecylguanidine Hydrochloride (DGH)

The inhalation aerosols of the DGH solutions were generated using an atomizer with a clean air flow of 250 L/min. For the acute inhalation exposure study, the rats were exposed to 7.8 mg/m^3^, 23 mg/m^3^, and 70 mg/m^3^ DGH for 4 h in the low, middle, and high exposure groups, respectively, and the control rats were exposed to clean air. In the acute toxicity test, all the rats in each group were sacrificed immediately after exposure. Temperature, humidity, air flow, and air pressure were monitored during the exposure using a Model VT3-X15 environmental controller (Sibata, Saitama, Japan). The concentration of DGH was analyzed by sampling the chamber air using a SIP-32L (Sibata, Saitama, Japan) and measuring the weight of the membranes (T60A20, Φ55). The mass median aerodynamic diameter (MMAD) and geometric standard deviation (GSD) of DGH in the exposure chamber were measured using an Andersen cascade impactor (AN-200, Sibata, Saitama, Japan).

### 2.7. Analysis of Blood

Hematology and serum biochemistry of ten rats in each group were analyzed, as described in previous studies [17,18]. Briefly, blood samples of the animals were drawn from the ventral aorta using a syringe, under anesthesia. The blood samples were collected into CBC bottles containing ethylenediaminetetraacetic acid (EDTA) (K2 EDTA, BD vacutainer) and analyzed within 15 min, using an automatic hematology LC-662G, Kyoto, Japan. The following parameters were determined: white blood cell count, red blood cell count, hemoglobin, hematocrit, mean corpuscular volume, mean corpuscular hemoglobin, mean corpuscular hemoglobin concentration, red blood cell volume distribution width, platelets, mean platelet volume, procalcitonin, and platelet distribution width. Blood samples were drawn from the ventral aorta using a syringe and collected in a serum vacutainer. The sera were collected by centrifugation at 2000 rpm for 10 min within 2 h after collection and stored at −80 °C in a freezer, prior to analysis. The following serum biochemistry parameters were determined using an automatic serum analyzer (Drichem4000, Fuji Photo Film, Tokyo, Japan): alkaline phosphatase, lactate dehydrogenase (LDH), glucose, total cholesterol, glutamic oxaloacetate transaminase, glutamic pyruvate transaminase, urea nitrogen in blood, calcium, inorganic phosphorus, gamma glutamyl transferase, albumin, total protein, creatinine, total bilirubin, sodium, potassium, and chlorine.

### 2.8. Analysis of Bronchoalveolar Lavage Fluid (BALF)

BALF was obtained from the right lung by lavage (twice) with 3 mL phosphate buffered saline (PBS, pH 7.4, magnesium, and calcium free). The BALF samples were centrifuged (1500 rpm, 4 °C, 10 min) and the cell-free supernatant of the first lavage was used for the determination of LDH, total protein, and inflammatory cytokine levels. LDH activity was measured using the EZ-LDH kit (DoGenBio, Seoul, Korea). Total protein content was quantified using a BCA protein assay kit (iNtRON Biotechnology, Seoul, Korea). To evaluate the inflammatory cytokine levels in BALF, tumor necrosis factor-alpha (TNF-α), interleukin-6 (IL-6), interleukin-1 beta (IL-1β), monocyte chemoattractant protein-1, and transforming growth factor-beta 1 (TGF-β1) were determined using a Quantikine^®^ enzyme-linked immunosorbent assay kit (R&D Systems, Minneapolis, MN, USA), according to the manufacturer’s instructions. Cells in all lavages were resuspended in PBS, and the total cell count in BALF was quantified using a Vi-Cell^®^ XR analyzer (Beckman Coulter, Brea, CA, USA). Differential cell counts were performed on cytospin preparations (Shadon, Pittsburgh, PA, USA), and the cells were stained with Diff-Quick (Fisher Scientific, Swedesboro, NJ, USA). Polymorphonuclear leukocytes (PMNs) in the BALF were counted using light microscopy (Olympus, Tokyo, Japan).

### 2.9. Histopathological Analysis

After the inhalation of DGH for 4 h, the animals were euthanized. After gross examination, the left lung and nasal cavity of the rats in the control and high-exposure groups were fixed in 10% neutral buffered formalin. The nasal cavity was decalcified with a decalcification solution and then cross-sectioned into four levels as follows: level 1 (posterior part of the upper incisors), level 2 (incisive papilla), level 3 (second palatine crest), level 4 (first upper molar teeth). Following routine tissue processing, the tissues were embedded in paraffin and sectioned into 3 μm thick sections. The tissue sections were stained with hematoxylin and eosin (H&E), and then examined under a light microscope (Olympus BX41, Tokyo, Japan). The lesions were graded according to severity by pathologists.

### 2.10. Statistical Analysis

All results are expressed as the mean ± standard error of mean. Means of different groups were compared using two-tailed unpaired Student’s t-tests or one-way analysis of variance (ANOVA, Tukey’s multiple comparison test) by GraphPad Prism version 5.0 software (GraphPad, La Jolla, CA, USA).

## 3. Results

### 3.1. Cytotoxicity of Guanidine-Based Chemicals

**Figure 1:**
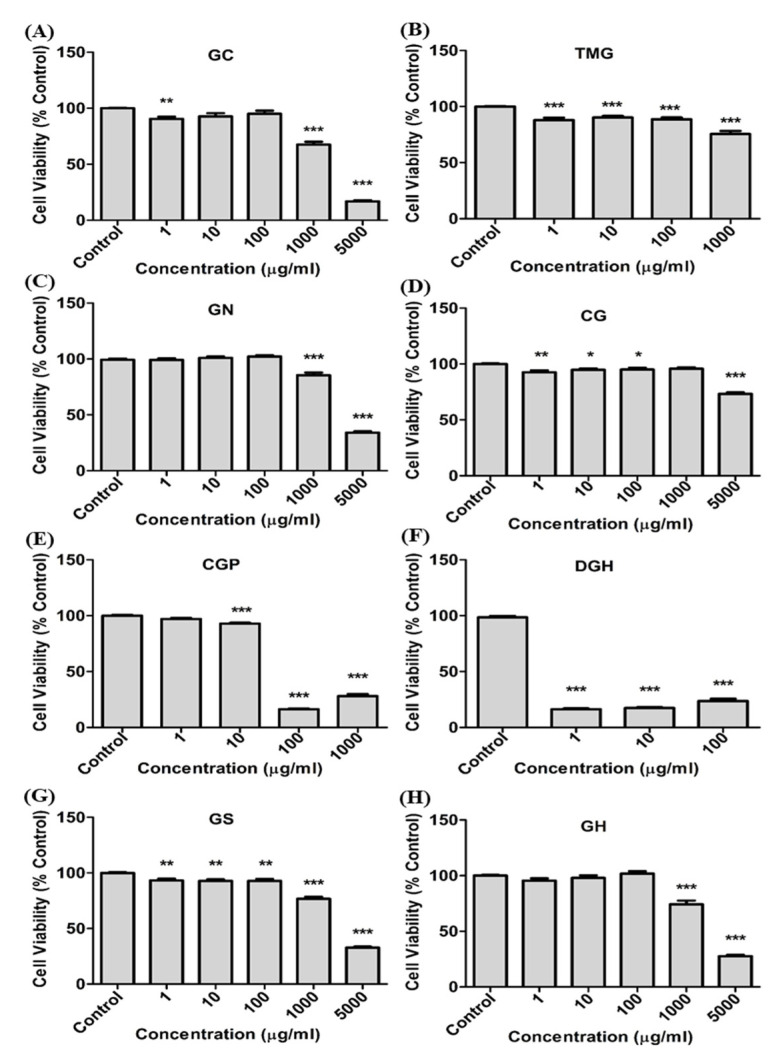
Cell viability (WST assay) of guanidine-based chemicals for cytotoxicity screening in human lung epithelial cells. (**A**) Guanidine carbonate (GC), (**B**) 1,1,3,3-Tetramethylguanidine (TMG), (**C**) Guanidine mononitrate (GN), (**D**) Cyanoguanidine (CG), (**E**) Cyanoguanidine polymer with ammonium chloride and formaldehyde (CGP), (**F**) Dodecylguanidine hydrochloride (DGH), (**G**) Guanidine sulfamate (GS), (**H**) Guanidine monohydrochloride (GH). A549 cells were exposed to each chemical for 24 h. (One-way ANOVA test, *, **, *** *p* < 0.05, 0.01, 0.001 vs. control).

**Figure 2:**
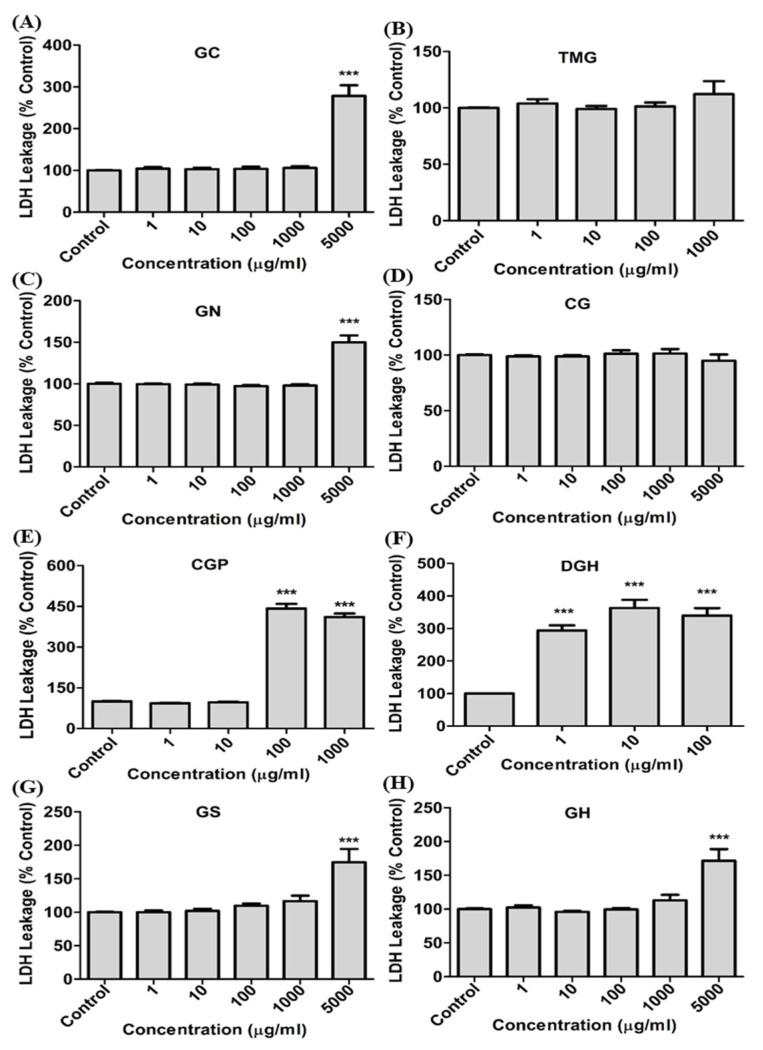
Cell membrane damage (lactate dehydrogenase [LDH] leakage) assay of guanidine-based chemicals in human lung epithelial cells. (**A**) Guanidine carbonate (GC), (**B**) 1,1,3,3-Tetramethylguanidine (TMG), (**C**) Guanidine mononitrate (GN), (**D**) Cyanoguanidine (CG), (**E**) Cyanoguanidine polymer with ammonium chloride and formaldehyde (CGP), (**F**) Dodecylguanidine hydrochloride (DGH), (**G**) Guanidine sulfamate (GS), (**H**) Guanidine monohydrochloride (GH). A549 cells were exposed to each chemical for 24 h. (One-way ANOVA test, *** *p* < 0.001 vs. control).

**Figure 3:**
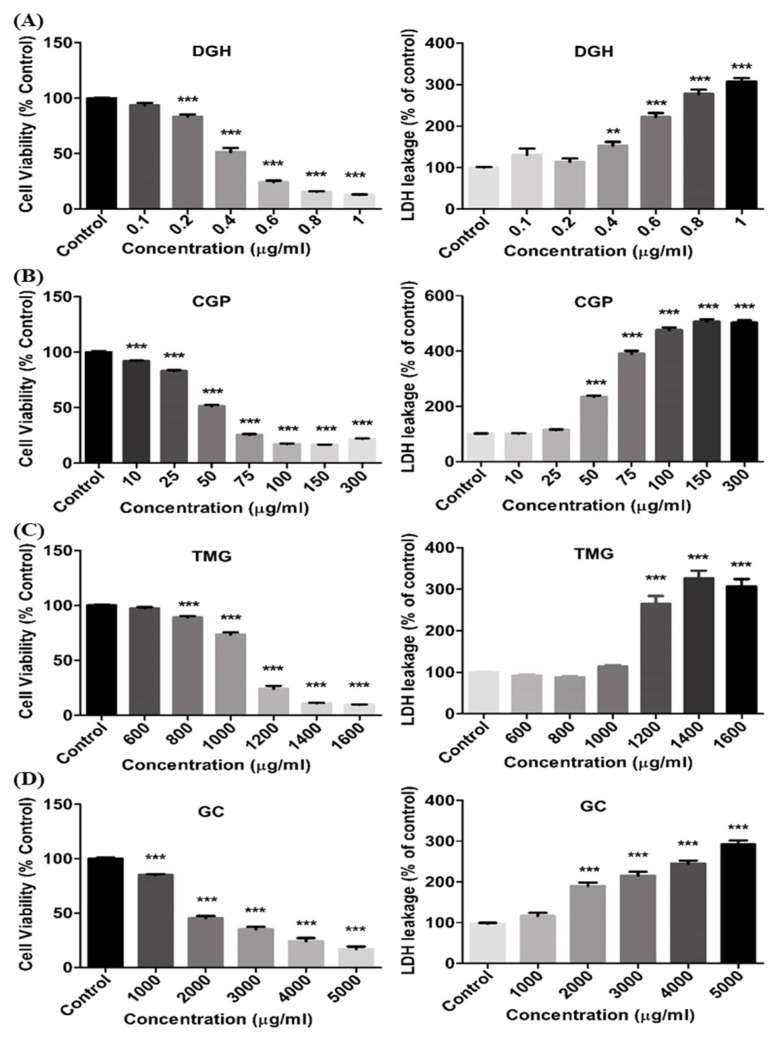
Cytotoxicity of 4 guanidine-based chemicals to human lung epithelial cells at a specific range of concentrations. (**A**) Dodecylguanidine hydrochloride (DGH), (**B**) Cyanoguanidine polymer with ammonium chloride and formaldehyde (CGP), (**C**) 1,1,3,3-Tetramethylguanidine (TMG), (**D**) Guanidine carbonate (GC). A549 cells were exposed to each chemical for 24 h. (One-way ANOVA test, **, *** *p* < 0.01, 0.001 vs. control).

The cytotoxicity of guanidine-based chemicals was evaluated in A549 cells. The cells were treated with guanidine-based chemicals (1–5000 μg/mL) and WST and LDH assays were performed. Exposure to guanidine-based chemicals resulted in a decrease in cell viability of A549 cells, as determined by the WST assay. In addition, results from the LDH assays showed an increased damage to cell membranes, in a dose-dependent manner (Figure 1 and Figure 2). DGH and CGP appeared to be more cytotoxic than other guanidine-based chemicals (Table 1). Cytotoxicity tests at specific range of concentration were performed for the four most toxic compounds to confirm accurate IC_50_ (Figure 3). These results showed that DGH and CGP showed higher cytotoxicity than the other eight compounds, with IC_50_ values of 0.39 and 49.6 μg/mL, respectively (Table 1).

### 3.2. Inhibition of Colony Formation of A549 cells

**Figure 4:**
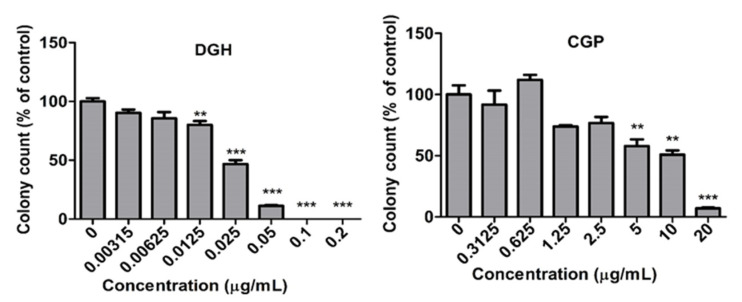
Colony formation of human alveolar (A549) cells treated with Dodecylguanidine hydrochloride (DGH) and Cyanoguanidine polymer with ammonium chloride and formaldehyde (CGP) A549 cells cultured at low density (200 cells/well) were exposed to CPG (0.3125–20 μg/mL) and DGH (0.00315–0.2 μg/mL) for 10 days. Mean ± SE. Student’s *t* test, **, *** *p* < 0.01, and 0.001, respectively, vs. control.

Clonogenic assays were performed for the two compounds that showed the highest cytotoxicity among the eight guanidine-based chemicals, DGH and CGP. A549 cells were treated with 0.00313–0.2 μg/mL DGH and 0.313–20 μg/mL CGP, and then incubated for 10 days. After 10 days, exposure to both DGH and CGP caused a dramatic decrease in the total number of colonies in a dose- dependent manner (Figure 4). DGH was found to be more toxic than CGP, and therefore, we decided to evaluate the pulmonary toxicity of DGH in rats.

### 3.3. Acute Inhalation of DGH

The actual exposure concentration of DGH was found to be 8.6 ± 0.41, 21.3 ± 0.83, and 68 ± 3.46 mg/m^3^ in the low, middle, and high exposure groups, respectively. The MMAD and GSD, as determined by the multistage impactor, were in the range of 1.2–1.38 μm, and 1.7–2.94, respectively, which was in accordance with the conditions of the OECD Test Guideline for Chemicals [19]. Body weight, hematology, and serum biochemistry showed no significant changes when compared to those of the control group. However, the spleen weight of male rats in the exposed groups was significantly decreased.

**Figure 5:**
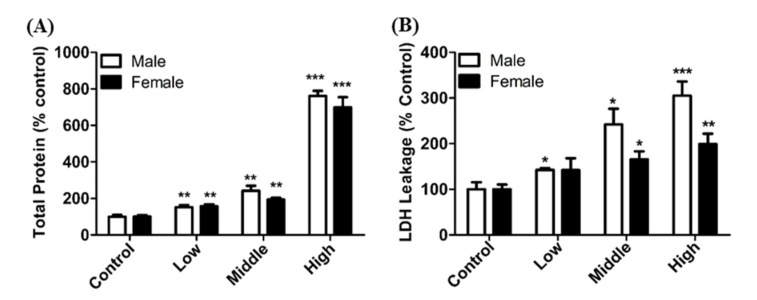
Pulmonary toxicity induced by Dodecylguanidine hydrochloride (DGH) in rats. (**A**) Total protein content in bronchoalveolar lavage fluid (BALF), (**B**) LDH activity in BALF. Male and female rats were exposed to aerosols of DGH for 4 h. Control; air-fresh, Low; 8.6 ± 0.41 mg/m3, Middle; 21.3 ± 0.83 mg/m^3^, and High; 68 ± 3.46 mg/m^3^. Each value represents mean ± SE (*n* = 5). Student’s *t* test, *, **, *** *p* < 0.05, 0.01, and 0.001, respectively, vs. control.

**Figure 6:**
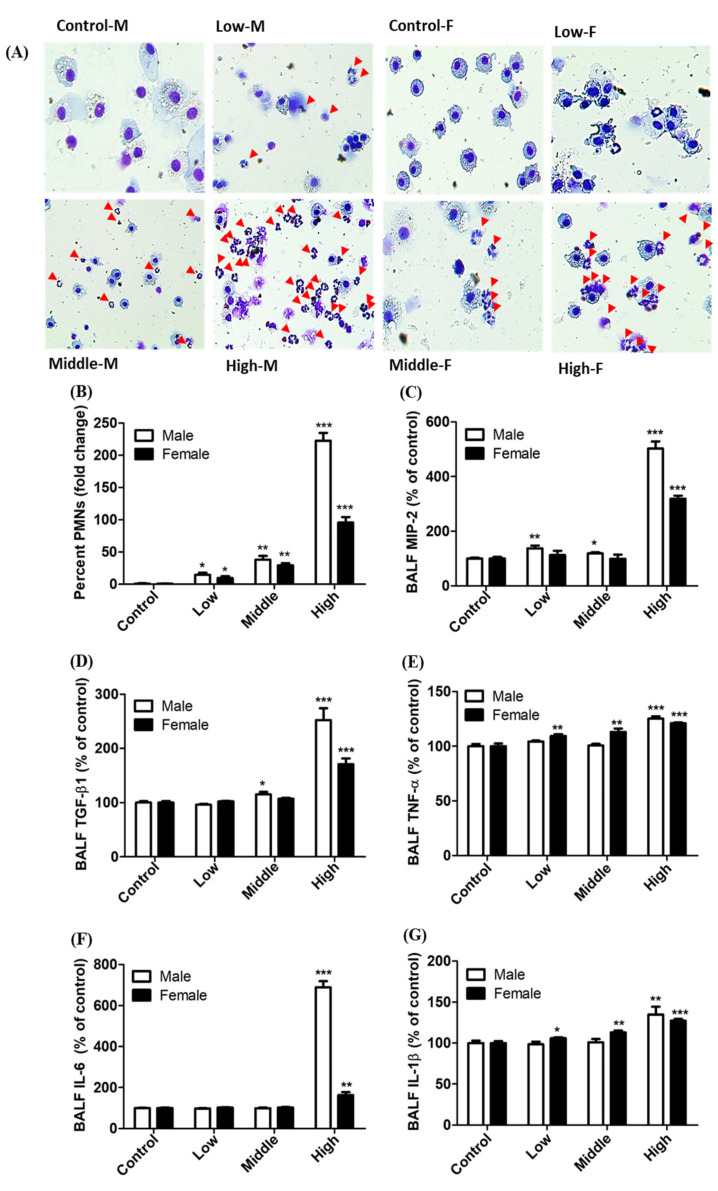
Pulmonary inflammation after inhalation of Dodecylguanidine hydrochloride (DGH) in rats. (**A**) Diff-quick staining of BALF cells, (**B**) Polymorphonuclear leukocytes (PMNs) in BALF, (**C**) MIP-2 in BALF, (**D**) TGF-β1 in BALF, (**E**) TNF-α in BALF, (**F**) IL-6 in BALF, (**G**) IL-1β in BALF. Male (M) and female (F) rats were inhaled with aerosol DGH for 4 h. Control; air-fresh, Low; 8.6 ± 0.41 mg/m^3^, Middle; 21.3 ± 0.83 mg/m^3^, and High; 68 ± 3.46 mg/m^3^. Arrow indicates PMNs. Each value represents mean ± SE (*n* = 5). Student’s *t* test, *, **, *** *p* < 0.05, 0.01, and 0.001, respectively, vs. control.

Acute inhalation of DGH resulted in a concentration-dependent induction of pulmonary toxicity, as was evident by the increase in total protein and LDH levels in the BALF analysis (Figure 5). These two representative pulmonary markers, total protein and LDH, have been used to analyze level of injury of lung cells and airway [20,21]. Furthermore, increase in the injury markers in male rats was more apparent than those in female rats in all the exposure groups. In addition, the number of PMNs in BALF in male rats was higher than that in female rats (Figure 6). The levels of pulmonary inflammatory cytokines, such as MIP-2, TGF-β1, IL-1β, TNF-α, and IL-6, in BALF were significantly higher in male rats than in female rats, exposed to DGH (Figure 6). These results showed gender differences in the expression of pulmonary inflammation after exposure to DGH.

**Figure 7:**
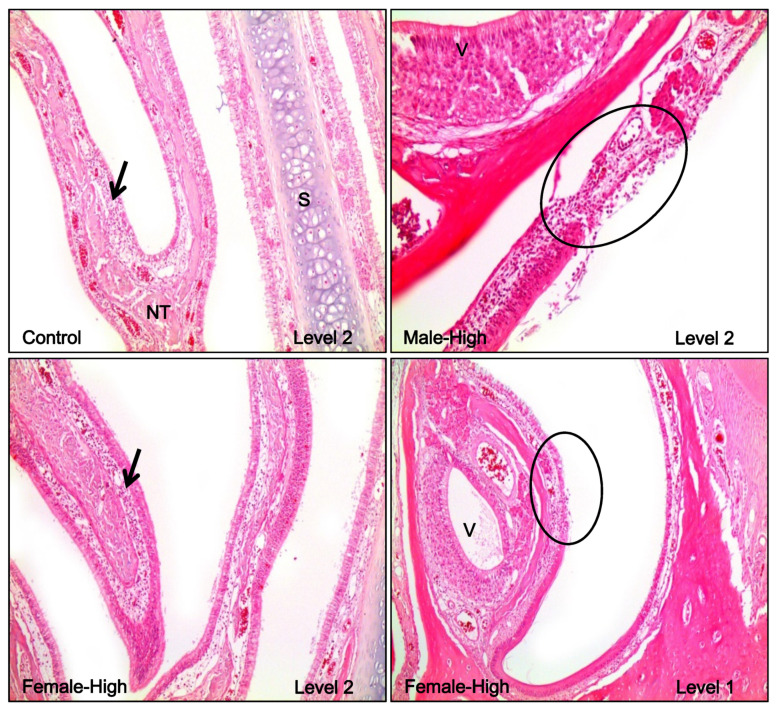
Histological features of the nasal cavity with pathological lesions in male and female rats in the acute inhalation study of Dodecylguanidine hydrochloride (DGH). Note the focal ulcerative acute inflammation at the transition area between the squamous and respiratory epithelial compartments of the ventral portion of nasal cavity (circles). In addition, note the focal infiltration of lymphocytes in the lamina propria and submucosa (arrows). NT, nasoturbinate; S, nasal septum; V, vomeronasal organ. H&E. Magnification, 100× except for 200× male-high.

**Figure 8:**
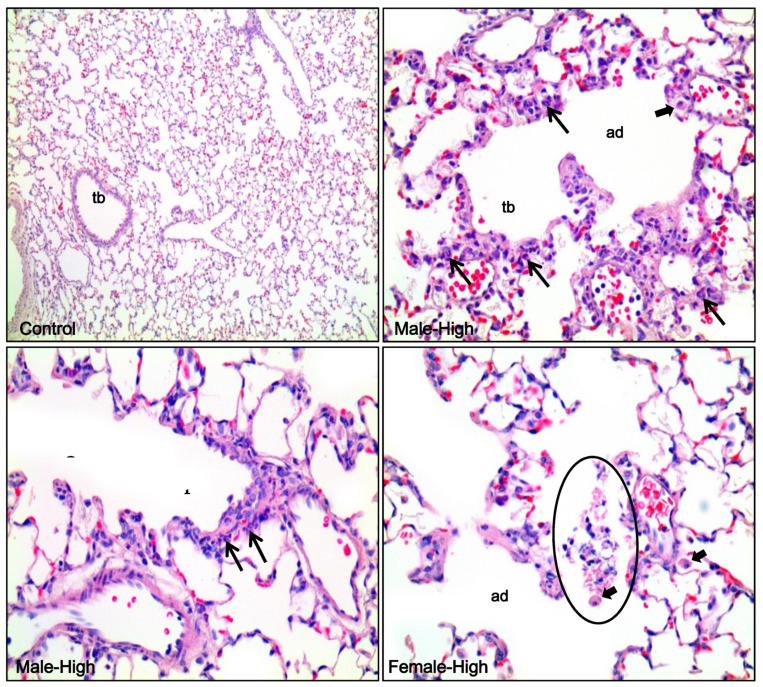
Histological features of lungs of male and female rats. No abnormal findings were observed in the control group. Note the infiltration of a small number of neutrophils (thin arrows) and alveolar macrophages (thick arrows) around terminal bronchioles (tb) and alveolar ducts (ad). In addition, note the intraluminal necrotic cell debris in the alveolar duct (circle). H&E. Magnification, 100× for control and 400× for others.

The summary of the histopathology of the nasal cavity and lung in rats after the acute inhalation of DGH is displayed in Table 2. Focal ulceration and acute inflammation were observed in one male and one female rat in the high exposure group, at the transition area between squamous epithelial and respiratory epithelial part of ventral portion in the level 1 and level 2 sections (Figure 7). This inflammatory lesion was not observed in the non-treated control group, and hence it was considered to be due to the effects of inhalation exposure to DGH. Focal submucosal infiltration of lymphocytes at levels 3 and 4 in the nasal cavity was found in the control and high exposure groups. This was believed not to be related to the inhalation of DGH because it also occurred in the control groups of both males and females, and the extent of severity was minimal. In the lungs of rats, no abnormal findings were observed in both the male and female control groups. However, in most of both male and female rats in the high exposure groups, infiltration of a small number of neutrophils and alveolar macrophages was noted around the terminal bronchioles and alveolar ducts. As a result, injury of alveolar epithelial cells in alveolar ducts was thought to be related to the inhalation exposure to DGH. Furthermore, specific abnormal findings, necrotic cell debris in the terminal bronchioles, were evident in a few rats (Figure 8).

## 4. Discussion

Guanidine-based chemicals, such as PHMG and PGH, have attracted considerable attention as antimicrobial compounds, owing to their high water solubility, excellent sterilization efficiency, and wide antimicrobial spectrum. Accordingly, synthetic guanidine-containing chemicals offer a wide range of possibilities and applications in various fields. Therefore, using these chemicals as a biocide has been a major trend in modern macromolecular chemistry [22,23,24,25,26,27].

We investigated the cytotoxicity and inhalation toxicity of the guanidine-based chemicals distributed in Korea, in order to address safety concerns related to substances with similar structures to PHMG and PGH. First, we performed cytotoxicity assays for eight guanidine-based chemicals, and then conducted acute inhalation toxicity studies for DGH, which was found to be the most toxic compound among them. DGH has been used as a guanidine-type cationic surfactant and antimicrobial biocide as well as a treatment material for paper that contacts with food, paint additives, and anti-bacterial treatment material of diapers [28]. Although toxicological information of DGH is limited, the Reregistration Eligibility Decision (RED) document for dodecylguanidine acetate (also known as dodine), published by the US Environmental Protection Agency, described that DGH and dodine are salts of the same chemical, have similar dissociation pattern, and are bioequivalent and toxicologically identical [29]. This document showed that the acute oral toxicity LD_50_ (rat) was 1931 mg/kg, dermal toxicity LD_50_ (rabbit) was >2000 mg/kg, and inhalation toxicity LC_50_ (rat, 1 h) was 1.05 mg/L for dodine. In vitro results using dodine as well as DGH were mostly related to genotoxicity or ecotoxicity studies, and cytotoxicity studies using lung cell lines have rarely been conducted. Thus, results from our study may be helpful in filling this data gap. Results from our in vitro study showed that DGH caused significant dose-dependent damage to the cell membrane, thereby decreasing cell viability. In addition, it had the highest cytotoxicity (IC_50_ of 0.39 μg/mL) among the eight guanidine-based chemicals. In the clonogenic assay, approximately 50% reduction in colony formation was induced at a DGH concentration as low as 0.025 μg/mL, when compared to the control. This suggested that the prolonged treatment effect of DGH as well as the effect of short-term exposure to A549 cells was higher than CGP.

Results from the acute inhalation study of DGH showed that no deaths occurred in the DGH (8.6–68 mg/m^3^) exposed groups. It was also observed that the organ weight of the spleen decreased significantly in the male exposure groups. Previous studies have reported that spleen weight changes may be due to various blood diseases, such as cirrhosis, hepatitis, pancreatic disease, and systemic infection [30]. Further research should include the investigation of a definite cause. LDH, an indicator of cytotoxicity, and total protein, an indicator of damage in cell membrane integrity, are useful biomarkers in determining lung tissue and pulmonary cell injury [31,32]. Our results showed that these biomarkers significantly increased in all the exposed groups compared to that in the control group. These results were consistent with the results from the cytotoxicity study. Male rats were found to have higher damage than female rats, both with respect to LDH and TP. Similar results were observed with respect to the changes in inflammatory factors.

Pulmonary inflammation is generally associated with lung damage caused by inhaled compounds [33,34]. When the respiratory system is exposed to foreign substances, increase of PMNs and rapid activation of macrophages are typical cellular changes in BALF as acute inflammatory symptom. It has been suggested that neutrophil influx plays a major role in inducing cytotoxicity during inflammation and increasing the permeability of the alveolar/capillary barrier [32]. Thus, the percentage of PMNs in BALF could be a useful indicator of pulmonary inflammatory responses. In the present study, a significant increase in PMNs was observed in all the exposed groups, and the number of PMNs increased in a concentration-dependent manner. The ratio of PMNs increased by more than 38.7-fold and 204-fold in the male rat groups exposed to DGH with middle (21.3 mg/m^3^) and high (68 mg/m^3^) concentrations, respectively. The ratio of PMNs in female rats also increased; however, the increase was not as high as that in male rats. A similar tendency was observed with respect to changes in the levels of inflammatory cytokines. Inflammatory cytokines initiate inflammatory responses and regulate host defense against pathogens that trigger innate immune responses [35]. In the present study, changes in inflammatory cytokines, such as MIP-2, TGF-β1, TNF-α, IL-6, and IL-1β, were observed in the exposure groups, which was similar to the results regarding PMNs, MIP-2, TGF-β1, and IL-6 showed higher expression levels in the exposure groups (about seven times that of IL-6 and about five times that of MIP-2 in male rats) compared to the control groups. IL-6 is a multifunctional cytokine that plays an important role in the response to infection or systemic inflammation, often referred to as ‘acute phase reactions’ [36]. It has been reported that the expression of protein in these acute phase reactions is mainly regulated by the action of IL-6 when severe damage to tissues occurs, and IL-6 is higher in males than in females [36,37,38]. Additionally, in the present study, elevated MIP-2 levels in male and female rats were closely associated with increased PMN recruitment into the lungs. MIP-2 plays a major role in neutrophil recruitment to the region of tissue damage, infection, and wound recovery [39,40]. BALF results, such as total protein, LDH, PMNs, and inflammatory cytokines, showed that males were more susceptible to DGH exposure than females.

Results from the histopathological examination showed that acute inflammatory changes were evident in the lung parenchyma, characterized by infiltration of neutrophils and macrophages around the terminal bronchioles and alveolar ducts. In the nasal cavity, focal ulcerative acute inflammation was observed in level 1 and level 2 areas located at the front of the nasal cavity. Although this pathological finding had a low incidence, it was considered to be related to DGH inhalation exposure because this lesion is not commonly observed under general conditions. Our results, including those from BALF analysis and histopathological examination, indicated DGH-related toxicities in the respiratory pathways. However, there was no death at the high concentration in this acute inhalation studies, therefore, we suggest that the LC_50_ for inhalation toxicity of DGH could be over 68 mg/m^3^, the highest concentration in this acute inhalation study. In future, to determine the inhalation toxicity of DGH more accurately, it would be necessary to conduct an inhalation study with longer exposure durations.

Cationic active biocides, such as quaternary ammonium compounds (QACs), guanidine derivatives, and amphoteric surfactants, have been widely used for a long time. Many biocides use high binding affinity with a containing cationic group to capture negatively charged bacteria, resulting in disruption of their membrane [41,42,43,44]. Having a highly positively charged part is the typical chemical characteristic of guanidine-based chemicals, and it is well known that positively charged parts of chemicals can penetrate the cell membrane easily, inducing systemic toxicity in vivo [45,46,47]. Therefore, the pulmonary toxicity of DGH in this study might be due to the property of positively charged guanidine.

In conclusion, the toxicity of DGH was classified assimilar to dodine because of its similar chemical structure and properties [29]. In the case of dodine, it has already been regulated by the government, classified as a toxic substance. Moreover, DGH is currently under control as it is registered as a biocide in the Republic of Korea. In the present study, we confirmed that DGH, which has relatively poor toxicity information compared to dodine, can induce clear pulmonary toxicity in SD rats and strong cytotoxicity in lung cells. Although only 4 h of inhalation exposure was performed, results from this study confirmed that DGH can cause lung injury and inflammation in SD rats. Moreover, our results suggest that exposure to DGH in the form of aerosols which might be generated when using consumer products containing DGH, could induce harmful pulmonary effects on human health. Therefore, further long-term inhalation toxicity studies on DGH need to be conducted.

## Figures and Tables

**Table 1:** Half inhibitory concentration by cell viability assay after exposure of guanidine-based chemicals.

CAS No.	Chemical Name.	IC_50_ (μg/mL)
13590-97-1	Dodecylguanidine hydrochloride (DGH)	0.39
55295-98-2	Cyanoguanidine polymer with ammonium chloride and formaldehyde (CGP)	49.6
80-70-6	1,1,3,3-Tetramethylguanidine (TMG)	1091
593-85-1	Guanidine carbonate (GC)	1822
50-01-1	Guanidine monohydrochloride (GH)	2373
50979-18-5	Guanidine sulfamate (GS)	2722
506-93-4	Guanidine mononitrate (GN)	3417
461-58-5	Cyanoguanidine (CG)	>5000

**Table 2:** Histopathology in Sprague-Dawley (SD) rats after acute inhalation of Dodecylguanidine hydrochloride (DGH).

Organ/Histopathology	Sex	Male		Female	
	Group	Control	High	Control	High
Nasal cavity	No. examined	5	5	5	5
Level 1					
No specific lesion		4(80.0)	4(80.0)	5(100)	4(80.0)
Cell infiltration, lymphocytes, focal		1(20.0)	0(0.00)	0(0.00)	0(0.00)
Grade: minimal		1	0	0	0
Acute inflammation with necrosis, focal		0(0.00)	1(20.0)	0(0.00)	1(20.0)
Grade: minimal		0	0	0	1
mild		0	1	0	0
Level 2					
No specific lesion		4(80.0)	4(80.0)	4(80.0)	5(100)
Cell infiltration, lymphocytes, focal		1(20.0)	0(0.00)	1(20.0)	0(0.00)
Grade: minimal		1	0	1	0
Acute inflammation with necrosis, focal		0(0.00)	1(20.0)	0(0.00)	0(0.00)
Grade: minimal		0	0	0	0
mild		0	1	0	0
Level 3					
No specific lesion		5(100)	3(60.0)	5(100)	4(80.0)
Cell infiltration, lymphocytes, focal		0(0.00)	2(40.0)	0(0.00)	1(20.0)
Grade: minimal		0	2	0	1
Level 4					
No specific lesion		4(80.0)	5(100)	5(100)	3(60.0)
Cell infiltration, lymphocytes, focal		1(20.0)	0(0.00)	0(0.00)	2(40.0)
Grade: minimal		1	0	0	2
Lung	No. examined	5	5	5	5
No specific lesion		5(100)	0(0.00)	5(100)	1(20.0)
Cell infiltration, terminal bronchiole/alveolar duct, neutrophils/macrophages		0(0.00)	5(100)	0(0.00)	3(60.0)
Grade: minimal		0	5	0	3
Intraluminal cell debris, terminal bronchiole/alveolar duct		0(0.00)	1(20.0)	0(0.00)	1(20.0)
Grades: minimal		0	1	0	1

The parentheses represent percentage of cases with the lesion in the total examined tissue number.
